# Efficient CRISPR/Cas9-assisted gene targeting enables rapid and precise genetic manipulation of mammalian neural stem cells

**DOI:** 10.1242/dev.140855

**Published:** 2017-02-15

**Authors:** Raul Bardini Bressan, Pooran Singh Dewari, Maria Kalantzaki, Ester Gangoso, Mantas Matjusaitis, Claudia Garcia-Diaz, Carla Blin, Vivien Grant, Harry Bulstrode, Sabine Gogolok, William C. Skarnes, Steven M. Pollard

**Affiliations:** 1 MRC Centre for Regenerative Medicine, University of Edinburgh, Edinburgh, UK; 2 Wellcome Trust Sanger Institute, Wellcome Trust Genome Campus, Cambridge, UK

**Keywords:** Neural stem cell, CRISPR/Cas9, Genome editing, Gene targeting, Epitope tagging, Homologous recombination, Glioblastoma, Transcription factor

## Abstract

Mammalian neural stem cell (NSC) lines provide a tractable model for discovery across stem cell and developmental biology, regenerative medicine and neuroscience. They can be derived from foetal or adult germinal tissues and continuously propagated *in vitro* as adherent monolayers. NSCs are clonally expandable, genetically stable, and easily transfectable – experimental attributes compatible with targeted genetic manipulations. However, gene targeting, which is crucial for functional studies of embryonic stem cells, has not been exploited to date in NSC lines. Here, we deploy CRISPR/Cas9 technology to demonstrate a variety of sophisticated genetic modifications via gene targeting in both mouse and human NSC lines, including: (1) efficient targeted transgene insertion at safe harbour loci (*Rosa26* and *AAVS1*); (2) biallelic knockout of neurodevelopmental transcription factor genes; (3) simple knock-in of epitope tags and fluorescent reporters (e.g. *Sox2-V5* and *Sox2-mCherry*); and (4) engineering of glioma mutations (*TP53* deletion; *H3F3A* point mutations). These resources and optimised methods enable facile and scalable genome editing in mammalian NSCs, providing significant new opportunities for functional genetic analysis.

## Abstract (precis)

**Summary:** Optimised strategies and protocols enable a range of complex genetic manipulations directly in mouse and human neural stem cell lines.

## INTRODUCTION

Targeted editing of endogenous genes via homologous recombination (HR) – termed gene targeting – has been widely deployed in studies of pluripotent stem cells, most notably mouse embryonic stem cells (ESCs) (Capecchi, 2005). Complex genetic manipulations in these cells is made possible by their inherent experimental attributes: they can be expanded to large numbers without genetic transformation; they are easily transfected, enabling efficient delivery of exogenous DNA; they can undergo HR; and, perhaps most importantly, they can be selectively propagated by clonal expansion in order to isolate defined genetic variants (Glaser et al., 2005). Such properties have underpinned a repertoire of genetic engineering technologies, including gene knockouts, conditional mutagenesis, and endogenous protein tagging. This has transformed our ability to explore mammalian gene function.

Similarly, the *in vitro* culture of neural stem cells (NSCs) and neural progenitor cells – of various distinct classes – has proven to be a valuable experimental approach for exploring molecular processes controlling self-renewal and differentiation across development, tissue homeostasis, and in disease models of the central nervous system (CNS) (Gage and Temple, 2013). NSC lines display molecular hallmarks of forebrain radial glia and can be readily established and expanded as adherent monolayers, either following *in vitro* differentiation of pluripotent cells, or more directly by primary culture of germinal tissues from the developing and adult mammalian CNS (Conti and Cattaneo, 2010; Conti et al., 2005; Pollard et al., 2006). These tissue stem cells can be routinely propagated and clonally expanded as primary stem cell lines in the absence of spontaneous differentiation and/or genetic transformation, thereby providing an experimentally tractable somatic stem cell model.

Genetically normal NSC lines have proven particularly useful in studies in which large numbers of tissue-restricted stem cells are needed, such as biochemical analyses (Engelen et al., 2011), transcriptome profiling (Johnson et al., 2008; Webb et al., 2013), genome-wide mapping of chromatin modifications, DNA methylation and transcription factor binding (Bernstein et al., 2006; Caren et al., 2015; Meissner et al., 2008; Mikkelsen et al., 2007), and chemical or genetic screening (Diamandis et al., 2007; Hubert et al., 2013). Importantly, malignant cells displaying phenotypic and functional properties analogous to NSCs can also be isolated from glioblastoma (GBM) patient samples using similar culture conditions (Pollard et al., 2009; Singh et al., 2004). This enables comparison of genetically normal NSCs with their malignant GBM counterparts, which has been helpful in defining tumour-specific vulnerabilities (Danovi et al., 2013; Ding et al., 2013; Hubert et al., 2013; Pollard et al., 2009).

Despite experimental attributes that are analogous to ESC cultures, targeted genetic manipulations directly in mammalian NSC lines have not yet been reported. Gene targeting has been notoriously difficult in most somatic cell types owing to the inefficiency of HR. However, recent improvements in design and production of customisable nucleases capable of introducing site-specific DNA double-strand breaks (DSBs) have provided increasingly refined and reliable ways to manipulate the mammalian genome (Mali et al., 2013). Once introduced, DSBs are able to trigger endogenous homology-directed repair (HDR) mechanisms, thereby increasing gene targeting efficiencies at a locus of interest when an exogenously introduced repair template is delivered. Alternatively, DSBs can be repaired through the error-prone non-homologous end-joining (NHEJ) pathway, which normally results in random insertion or deletion (indel) mutations (Hsu et al., 2014). Therefore, in addition to facilitating gene targeting by HR, DSBs result in site-specific mutagenesis.

Among the different platforms described for introduction of site-specific DSBs in eukaryotic cells, the clustered regularly interspaced short palindromic repeats (CRISPR)/Cas9 system has emerged as the favoured technology (Hsu et al., 2014). Derived from the prokaryotic adaptive immune system, this consists of an RNA-guided endonuclease (Cas9) able to generate DSBs efficiently using Watson–Crick base pairing to identify target DNA (Jinek et al., 2012). CRISPR/Cas9 has been adapted for mammalian genome editing purposes through human codon optimisation of Cas9 and generation of chimeric single guide RNAs (sgRNAs) (Mali et al., 2013). The Cas9 has also been further modified by mutation of one of the two independent nuclease domains in order to generate a nickase variant (Cas9n), which provides greater target specificity when used with a pair of strand-specific sgRNAs (Ran et al., 2013).

Here, we exploited the CRISPR/Cas9 technology to demonstrate complex and precise genetic manipulations in both mouse and human NSC lines. We find that CRISPR/Cas9-assisted gene targeting in NSCs is highly efficient, easy to implement, and scalable. Optimised strategies and protocols were developed to support a range of targeted genetic manipulations, such as gene knockouts, knock-ins of epitope tags and fluorescent reporters, and delivery of disease-relevant mutations. As an example of the potential of this new technology, we focused our efforts on genes encoding neurodevelopmental transcription factors, given the wide interest in these across stem cell biology, reprogramming, regenerative medicine and neuro-oncology.

## RESULTS

### Mouse and human NSC cultures can undergo gene targeting via Cas9-assisted homologous recombination at ‘safe-harbour’ loci

**Fig. 1.**
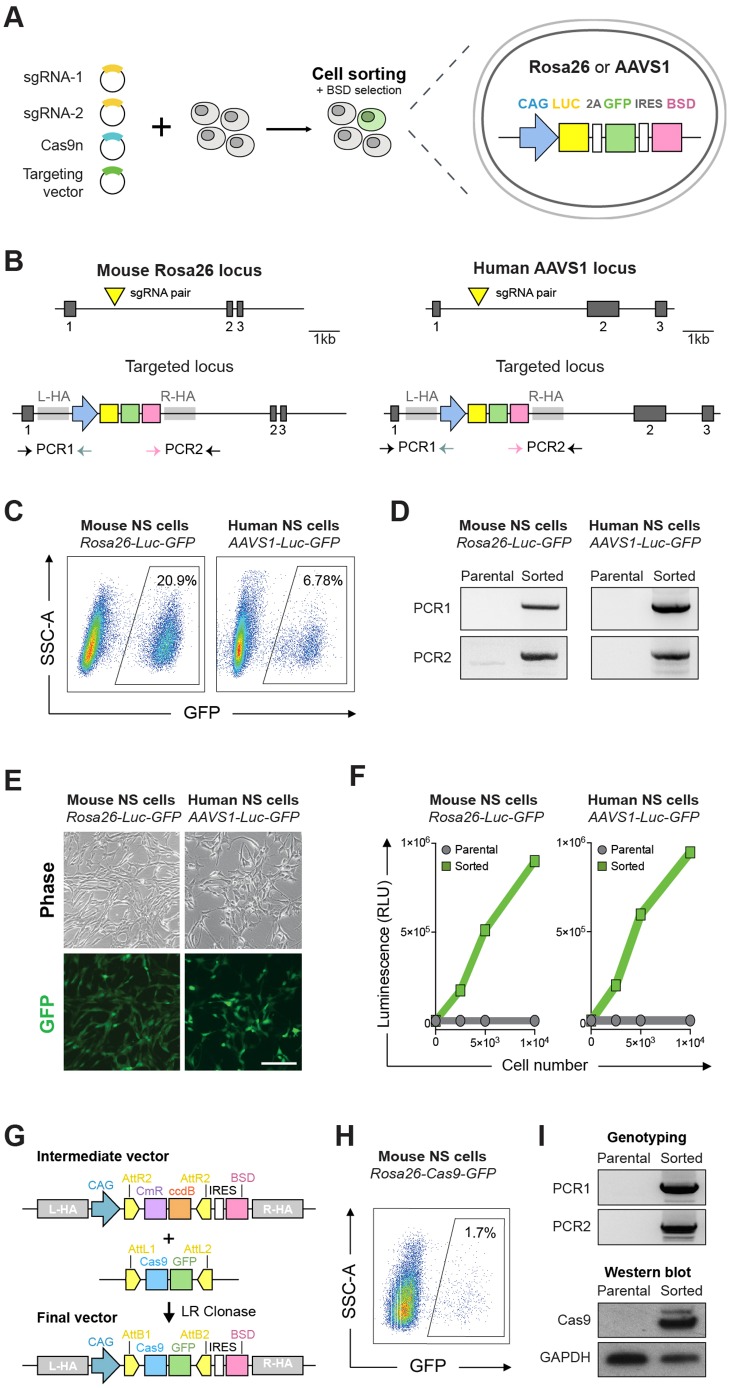
**Mouse and human NSCs are amenable to CRISPR/Cas9-mediated gene targeting.** (A) Schematic of the experimental strategy for generating constitutive Luciferase (Luc)-GFP expressing mouse and human NSCs by gene targeting at the safe harbour loci *Rosa26* or *AAVS1*. Targeting vectors contained the CAG-Luc-2A-GFP tethered by an IRES to a blasticidin resistance cassette (BSD); the expression cassettes were flanked by ∼1 kb long homology arms. (B) Schematic depiction of the targeting strategy for the *Rosa26* (left) and *AAVS1* loci (right). Exons are shown as dark grey blocks. Light grey rectangles indicate the location of the homology arms flanking the expression cassette (L-HA, left homology arm; R-HA, right homology arm). CRISPR sgRNA target sites are indicated with yellow triangles. Horizontal arrows indicate genotyping PCR primers used to confirm on-target integration of the expression cassette. (C) Using FACS, targeted cells were enriched on the basis of GFP expression after blastidicin selection. Wild-type non-transfected mouse and human NSCs were used as a control to set gates for cell sorting. SSC, side scatter. (D) PCR-based genotyping using primer sets 1 and 2 (depicted in A) confirmed correct targeted integration of the CAG-Luc-2A-GFP cassette into the *Rosa26* and *AAVS1* loci. Non-transfected parental cells were used as negative control for the genotyping. (E) Representative live phase contrast and wide-field fluorescence microscopy images of sorted GFP-positive mouse and human NSCs. Scale bar: 100 μm. (F) Luciferase levels were determined using a microplate reader and confirmed functionality of the targeted cassette in both mouse and human cells. (G) Schematic of the Gateway cloning-based strategy for repurposing targeting vectors with different cassettes of interest. In the example shown, the Luc-2A-GFP in the *Rosa26* targeting vector is replaced by a Cas9-GFP expression cassette via LR Gateway cloning. (H) Mouse NSCs were transfected with the *Rosa26* Cas9-GFP targeting vector and enriched by FACS on the basis of GFP expression. (I) PCR-based genotyping (top) confirms correct insertion of Cas9-GFP expression cassette at the *Rosa26* locus; western immunoblotting (bottom) confirms high levels of Cas9 protein expression in a clonal NSC line derived from the GFP-sorted cells.

Genomic safe harbours are known to undergo efficient HR in ESCs and induced pluripotent stem cells (iPSCs) and enable predictable expression of exogenous DNA elements across various cell types. To determine first whether mouse and human NSCs were amenable to Cas9-assisted gene targeting, we focused on the widely used safe harbour loci mouse *Rosa26* and human *AAVS1.* Targeting vectors with 1 kb homology arms flanking a constitutive Luciferase-2A-GFP-IRES-BSD expression cassette were produced and tested in an adult mouse NSC line (ANS4) and a human foetal NSC line (U3) (Fig. 1A,B; Fig. S1). Two matching CRISPR sgRNAs were designed to lie in close proximity and target opposite strands of each locus and could therefore be used as a pair with the Cas9n.

Transient plasmid transfection of Cas9n and sgRNA pairs was performed and GFP-expressing cells were isolated using fluorescence-activated cell sorting (FACS) 10-15 days after transfection and drug selection (Fig. 1B,C). PCR-based genotyping of the sorted population indicated successful targeted insertion of the expression cassette into *Rosa26* and *AAVS1* loci, and the majority of cells stably expressed high levels of GFP (>80%, visualised by microscopy) and luciferase (Fig. 1D-F). To determine the precise efficiency of correct insertion, clonal lines were generated from the sorted population using improved colony formation conditions (see Materials and Methods) and PCR genotyped (Fig. S2A). We achieved targeting efficiencies of ∼23% (11/48) and ∼16% (3/19) in mouse and human NSCs, respectively. Similar efficiency was achieved in a freshly derived foetal forebrain mouse NSC line (FNS2; Fig. S2B). Further PCR screening did not detect vector backbone sequences and qPCR copy number analysis indicated single copy integration in the majority of correctly targeted *Rosa26* clones (Fig. S2C,D). Genome-edited clonal lines proliferated normally, and uniformly expressed GFP and the known NSC markers nestin and Sox2. Diploid karyotype as well as glial and neuronal potential was maintained after continuous *in vitro* expansion (Fig. S2E,F).

Both *Rosa26*-Luc-GFP and *AAVS1*-Luc-GFP targeting vectors were constructed using intermediate vectors compatible with the Gateway cloning system (Fig. S3A). This strategy enables straightforward exchange of alternative cargos, which we exemplified by the generation a CAG promoter-driven Cas9-2A-GFP vector for targeting the *Rosa26* locus (Fig. 1G). Delivery of this construct into mouse NSCs enabled facile isolation of NSCs constitutively expressing Cas9 (termed CAS9-NSCs; used later in this study) using FACS (Fig. 1H,I). A similar strategy for targeting *AAVS1* also enabled rapid generation of Tet-inducible-GFP-expressing human NSCs (Fig. S3B-D).

Altogether, these results demonstrate that both mouse and human NSCs can undergo Cas9-assisted gene targeting and that promoter-driven expression cassettes can be efficiently knocked into safe harbour loci by HR. This has practical value, as it circumvents two issues – insertional mutagenesis and transgene silencing – that are often associated with conventional transgenic approaches (e.g. viral vectors or transposase delivery systems).

### Efficient biallelic disruption of *Olig2* in mouse NSCs using Cas9-induced NHEJ

Targeted inactivation of endogenous genes via deletion or mutation of coding sequences is the standard reverse genetics used to define gene function. Unlike RNAi technologies, loss of the gene product is unequivocal and permanent, and risks of undesirable non-specific effects are minimised. We therefore tested whether the CRISPR/Cas9 system could be used to disrupt gene expression efficiently in mouse NSC lines by site-specific mutagenesis.

**Fig. 2.**
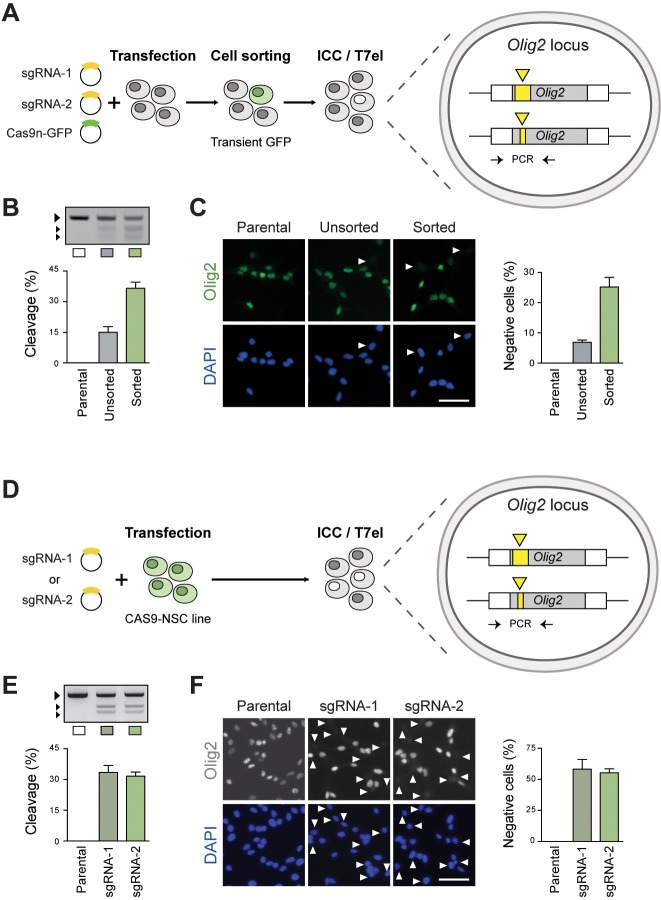
***Olig2* knockout in mouse NSCs via CRISPR/Cas9-induced NHEJ repair.** (A) Experimental strategy for *Olig2* deletion in wild-type (WT) mouse NSCs using transient plasmid delivery. Cells were transiently transfected with the CRISPR sgRNA pair (target site shown as yellow triangles) together with a Cas9n-2A-GFP plasmid. Transfected cells were enriched by FACS on the basis of GFP expression. (B) Generation of indel mutations in the transfected cells was assessed using a T7EI cleavage assay. Larger arrow indicates the predicted WT/uncleaved PCR product; smaller arrows indicate T7EI-cleaved fragments used to estimate indel frequency. (C) ICC was used to determine the frequency of Olig2-negative cells, which result from biallelic frameshift mutations (white arrowheads). Graph shows percentage of Olig2-negative cells relative to total DAPI-stained nuclei. Scale bar: 50 µm. (D,E) Experimental strategy for *Olig2* deletion in the CAS9 NSC line. Cells were transfected with the two CRISPR sgRNAs individually (D) and cleavage at target site confirmed by the T7E1 assay (E). (F) Efficiency of biallelic knockout was quantified by ICC for Olig2. Scale bar: 50 µm.

For this purpose, we focused on *Olig2*, a known NSC transcriptional regulator that is highly expressed in mouse NSC lines (Ligon et al., 2007). A pair of sgRNAs targeting the *Olig2* coding sequence and Cas9n-2A-GFP expression plasmids were transiently delivered into mouse NSCs, and successful transfectants were harvested by FACS (Fig. 2A). Formation of indel mutations around the sgRNA target sites was confirmed using the T7 endonuclease I (T7EI) assay (Fig. 2B). The frequency of biallelic mutations that result in loss of the Olig2 protein product was assessed by immunocytochemistry (ICC) with a specific Olig2 antibody. Using this strategy, we observed biallelic inactivation efficiencies of ∼7% in unsorted cells, and up to ∼25% in the sorted fraction (Fig. 2B,C). A similar frequency of Olig2-negative colonies was observed when the transfected cells were clonally expanded (32%, *n*=25; not shown). Similar efficiencies could be achieved using the independent foetal forebrain NSC line FNS2 (Fig. S4A).

Recent evidence suggests that Cas9 off-target cleavage is not as pervasive as initially feared and can be minimised using appropriate sgRNA design rules (Kim et al., 2016). We therefore tested the wild-type Cas9 (WTCas9-2A-GFP), rather than the nickase, as this provides a more convenient strategy. The two sgRNAs (predicted to target unique genomic sites; see Materials and Methods) were delivered separately and, following FACS enrichment, we noted that significantly higher efficiencies could be achieved to the Cas9n (Fig. S4B). Thus, both Cas9 and Cas9n can work effectively, with the latter being useful if unique gRNAs are unavailable.

We next delivered the individual sgRNA plasmids into our newly generated CAS9-NSC line to test whether even higher levels of mutations were possible (Fig. 2D). The T7EI assay indicated indel formation around the predicted cutting site, thereby confirming the activity of the constitutively expressed Cas9 (Fig. 2E). Remarkably, using this approach we achieve knockout efficiencies of around >50% for each sgRNA (Fig. 2E,F). Altogether, these results demonstrate the power of CRISPR/Cas9 to enable efficient biallelic disruption of *Olig2* via NHEJ in mouse NSCs. This can be achieved either by transient plasmid delivery of Cas9 or with increased efficiency using the constitutive Cas9-expressing NSC line.

### Efficient generation of *Olig2* knockout NSC lines using CRISPR/Cas9-assisted gene targeting

Despite the value in generating random indels by NHEJ, a more precise and flexible approach to manipulating endogenous genes is provided by HR-based gene targeting. This offers complete control over the type of allele to be generated and enables removal or replacement of any desired sequence. To test whether this was possible in mouse NSCs, we employed a gene targeting strategy recently developed for human iPSCs (W.C.S., unpublished). A selectable marker is used to replace a target exon of interest, thereby enabling enrichment for correctly targeted cells; these then emerge as discrete resistant colonies that can be selectively propagated and genotyped. Provided that biallelic cleavage is possible and DSBs are likely to be repaired more frequently by NHEJ than by HDR mechanisms, we anticipated that the most frequent type of editing event would be targeted replacement of one allele with the selectable marker, and generation of indels at the other. Thus, biallelic loss-of-function mutations might emerge from a single round of transfection and selection. Importantly, this approach does not require cell sorting or Cas9-expressing transgenic lines, and genotyping of clones is simplified by the fact that the indel-containing allele can be directly sequenced from the PCR reaction.

**Fig. 3.**
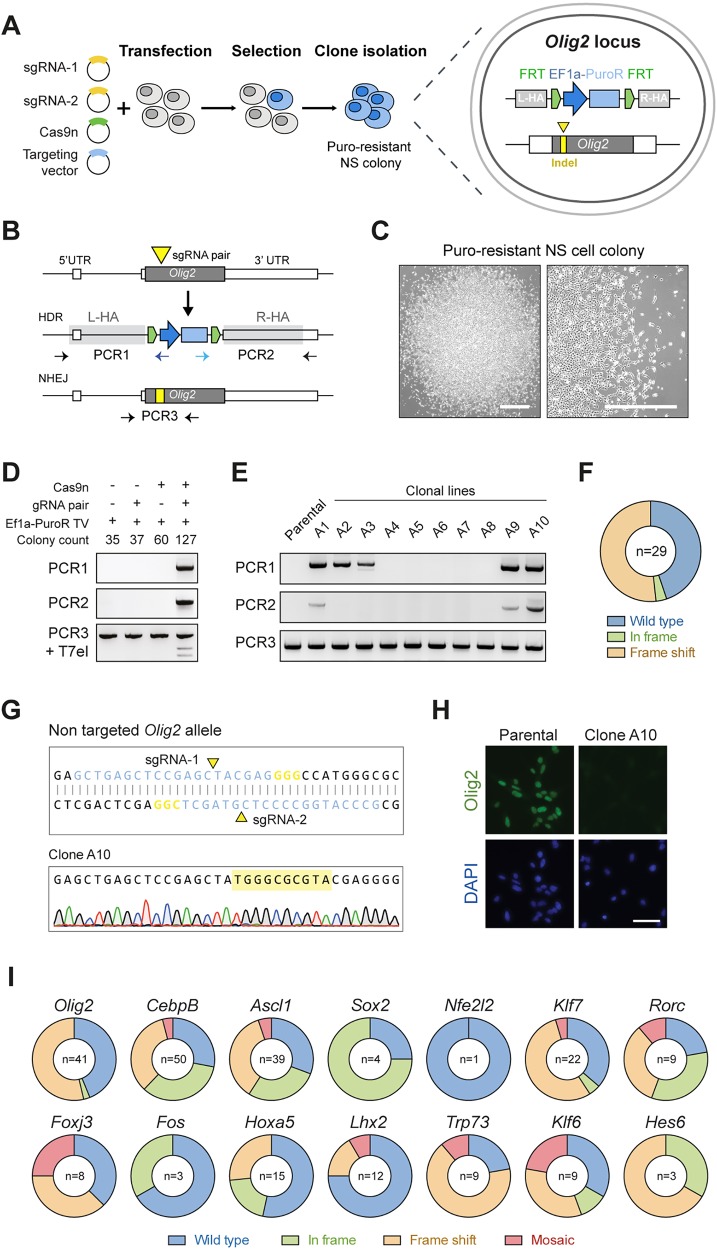
**CRISPR/Cas9-mediated homologous recombination enables facile knockout of transcription factor genes in mouse NSCs.** (A) Schematic of the experimental strategy to knock out *Olig2* via CRISPR/Cas9-assisted gene targeting. Targeted cells are enriched by puromycin selection and should emerge as discrete NSC colonies. Biallelic knockout clones are expected to have one allele replaced by the Ef1α-PuroR cassette and the second allele damaged by an indel mutation (yellow rectangle) at the CRISPR sgRNA cutting site (yellow triangle). (B) Representation of the mouse *Olig2* locus (top), and predicted targeted alleles following HDR (middle) or NHEJ (bottom). *Olig2* coding sequence is shown in dark grey. Adjacent white rectangles represent untranslated regions (5′ and 3′ UTR). PCR genotyping (PCR1 and 2, left and right arms, respectively) were designed with primers within the Ef1α-PuroR cassette and outside of the homology arms (light grey rectangles). PCR3 product could be used in a T7EI assay or Sanger sequencing to confirm the presence of indels in the NHEJ allele. (C) Representative phase contrast image of exemplar puromycin-resistant NSC colony, which emerged after transfection with Olig2 targeting vector. Scale bars: 200 µm. (D) PCR genotyping of pooled puromycin-resistant colonies after transfection with Olig2 targeting vector (Ef1α-PuroR TV) alone or in combination with CRISPR Cas9 nickase and/or sgRNA pair. PCR1 and PCR2 were used to confirm correct HR event at *Olig2* locus (top and middle); T7EI assay for checking NHEJ-mediated damaged at the sgRNA targeted site (bottom). (E) Representative genotyping PCR results of ten puromycin-resistant clones, picked and expanded as clonal lines following transfection with targeting vector and CRISPR/Cas9 components. (F) Summary of types of indel mutations identified by Sanger sequencing of PCR3 product (shown in B) in correctly targeted *Olig2* clones. Wild type, no indel; in frame, 3N; frame shift, 3N+1 or 3N+2. (G) Representative Sanger sequencing trace of one *Olig2* targeted clone showing 10 bp insertion within the remaining *Olig2* coding exon. (H) ICC in wild-type parental cells and Olig2 clone harbouring the 10 bp insertion confirms complete ablation of the Olig2 protein. Scale bar: 50 µm. (I) Scale-up of the same strategy used for *Olig2* in 14 other transcription factors and frequency of indel types present on the second allele of correctly targeted clones. Mosaics contained more than one indel within the same clone and were identified by mixed Sanger sequencing traces (see Fig. S5B).

We first focused on the mouse NSC regulator *Olig2*, attempting to replace its single coding exon with an Ef1α-puromycin (Ef1α-PuroR) selection cassette and assessing the presence of indel mutations on the second allele (Fig. 3A,B). The sgRNA pair described above was used together with a newly generated targeting vector with 1 kb long homology arms flanking the Ef1α-PuroR cassette. This was generated via production of a Gateway cloning compatible intermediate vector, as described for *Rosa26* and *AAVS1* vectors.

Mouse NSCs were transfected with the circularised targeting vector alone or in combination with Cas9n/sgRNAs plasmids, and treated with puromycin. Resistant NSC colonies emerged within 7 days (Fig. 3C), and PCR genotyping of pooled colonies was used to verify correct targeting at the *Olig2* locus (Fig. 3D). Importantly, correct replacement of the *Olig2* coding sequence by the Ef1α-PuroR cassette was only achieved when both the Cas9n and sgRNAs were co-transfected, thus confirming the need for the CRISPR/Cas9 system to achieve efficient HR in NSCs (Fig. 3D). As anticipated, using a T7EI assay we also observed formation of indels around the sgRNA cutting site, which are likely associated with NHEJ events within the non-targeted allele (Fig. 3D).

To determine targeting efficiencies and the precise status of each allele, puromycin-resistant colonies were picked and expanded as clonal NSC lines. PCR-based genotyping confirmed correct targeting at the *Olig2* locus in ∼26% of the screened clones (*n*=112; Fig. 3E). Biallelic replacement of the *Olig2* coding exon was not observed, as PCR with internal primers was in all cases able to amplify the non-targeted allele (Fig. 3E). Nevertheless, Sanger sequencing of the PCR products revealed a remarkably high frequency of indel mutations within the remaining allele of correctly targeted clones (*n*=16/29; Fig. 3F,G), thereby demonstrating the value of the strategy for efficient generation of biallelic mutants. Complete ablation of Olig2 protein levels was confirmed by ICC and western blot (WB) in the clones harbouring frame-shifting indels (i.e. 3N+1 or 3N+2) (Fig. 3G,H; Fig. S5A). The edited clonal lines were diploid, maintained NSC morphology and nestin expression, and proliferated similarly to parental controls (Fig. S5B). qPCR copy number analysis indicated single insertion of the Ef1α-Puro cassette in the majority of correctly targeted clones (Fig. S6A).

We also knocked out *Olig2* by gene targeting in a PDGFRα-H2B-GFP reporter NSC line (PG1-1) derived from a previously generated mouse strain (Hamilton et al., 2003). PDGFRα is a marker of oligodendrocyte progenitor cells and is activated as NSCs undergo differentiation. Puromocyin-resistant colonies were generated and, consistent with its known role, we found that Olig2-negative NSC colonies failed to give rise to GFP-positive oligodendrocyte precursor cells when triggered to differentiate (Fig. S7).

Together, these data demonstrate that generation of NSC lines with biallelic loss of function by gene targeting is highly efficient in mouse NSC lines. Remarkably, this can be achieved with only a single round of transfection and screening of a handful of clones, thereby greatly simplifying reverse genetics in NSCs.

### A scalable strategy for generating mutant alleles in mouse NSCs using CRISPR/Cas9-assisted gene targeting

To determine whether the observed high knockout efficiency was unique to *Olig2* we next assessed gene targeting for a further three neurodevelopmental transcription factor genes: *Cebpb*, *Ascl1* (also known as *Mash1*) and *Sox2* (Carro et al., 2010; Castro et al., 2011; Gómez-López et al., 2011). The coding sequences of these genes also lie within a single exon, thereby facilitating their complete ablation. Utilising the same gene-targeting strategy and delivery methods as for *Olig2* (Cas9n plus Ef1α-PuroR vector), an even higher targeting efficiency was achieved for *Cebpb* (∼57%; *n*=88). The majority of correctly targeted clones also harboured indel mutations at the non-targeted allele (36/50; biallelic mutation efficiency 72%). By comparison, for *Ascl1* we observed lower targeting efficiency (13.6%) and frequency of biallelic mutations (1.5%; *n*=66). However, simply switching to wild-type Cas9 significantly increased the frequency of *Ascl1* targeted clones and presence of indels within their second alleles (∼35 and ∼30%, respectively; *n*=86) (Table 1; Fig. 3I). Approximately 70% of correctly targeted *Cebpb* and *Ascl1* clones showed single-copy transgene integration as confirmed by qPCR copy number analysis (Fig. S6A).

**Table 1.**
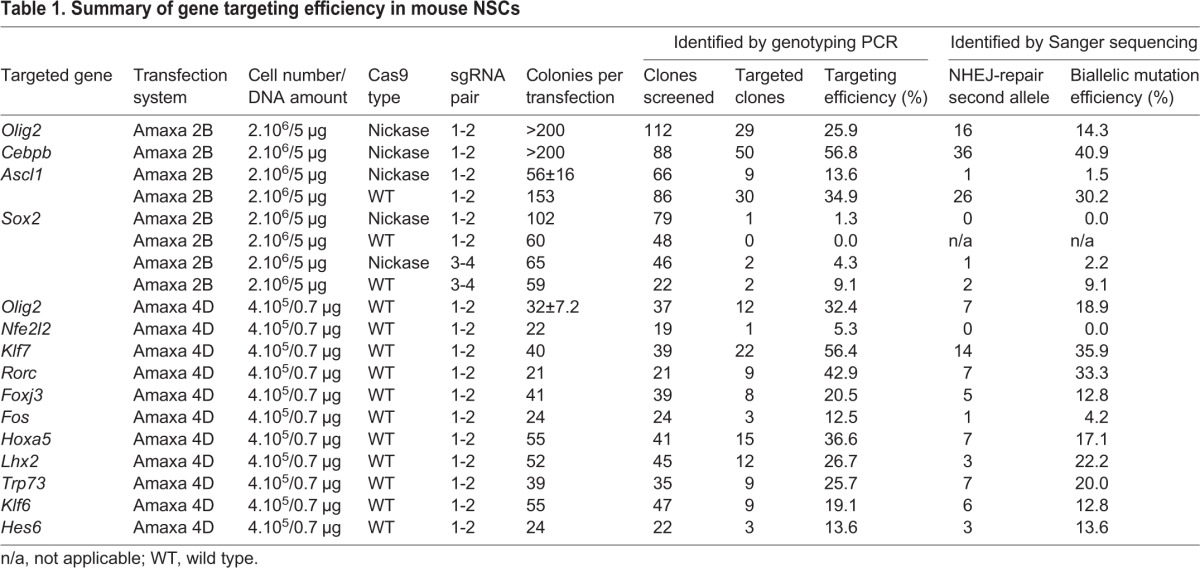
**Summary of gene targeting efficiency in mouse NSCs**

*Sox2* is required for *in vitro* NSC self-renewal (Gómez-López et al., 2011), and therefore recovery of expandable NSC clones with biallelic mutations and consequent loss of function should not be possible. To achieve targeting at the *Sox2* locus, we tested sgRNA pairs lying near to either the start or the stop codon (sgRNA pair 1-2 and 3-4, respectively) in combination with Cas9n or wild-type Cas9. As anticipated, only a limited number of puromycin-resistant NSC colonies emerged, and low targeting efficiencies ranging from ∼1.3% to ∼9.0% (*n*=79 and *n*=22, respectively) were achieved (Table 1). Importantly, biallelic knockout was not observed. The recovered targeted clones contained no damage or only small in-frame indels on the second allele (Fig. 3I; Fig. S8), confirming the essential role of *Sox2* in NSC colony formation. Failure to recover loss-of-function mutations on the NHEJ-damaged allele thereby provides unbiased functional genetic evidence that a gene is necessary for NSC self-renewal.

To enable medium throughput experiments, we optimised conditions to allow parallel transfection of 16 samples using reduced cell numbers and DNA amount with the Amaxa 4D system (Lonza) (see Materials and Methods). We designed and built targeting vectors and matched sgRNA pairs to knock out a set of 16 candidate regulators of NSC self-renewal, chosen as potential *Sox2* downstream transcriptional targets (Lodato et al., 2013; S.M.P. and H.B., unpublished). Wild-type Cas9 was used in these experiments to maximise recovery of targeted clones. We found that efficient targeting can be achieved using only 400,000 cells and <1 µg of plasmid DNA (Fig. S9).

Using these optimised conditions, we targeted 16 genes in a single experiment, with mutant clones obtained, expanded and genotyped within 3 weeks. Eleven of these were successfully targeted (*Olig2*, *Nfe2l2*, *Klf7*, *Rorc*, *Foxj3*, *Fos*, *Hoxa5*, *Lhx2*, *Trp73*, *Klf6*, *Hes6*), with efficiencies ranging from 5 to 56% and loss-of-function mutants recovered in most of the cases (Table 1; Fig. 3I; Fig. S8). Thus, biallelic knockouts can be routinely generated by Cas9-assisted gene targeting in an efficient and scalable manner in mouse NSCs.

### Knock-in of a fluorescent reporter to generate *Sox2*-*mCherry* reporter NSCs

Knock-in of fluorescent reporters to generate in-frame fusion proteins provides a useful experimental approach to monitor levels and localisation of a specific gene product. Such a strategy has been widely deployed in ESCs and iPSCs to allow real-time observation of gene-expression dynamics, cell-lineage tracing, and isolation of a specific cell population of interest from differentiating cultures or embryos (Goulburn et al., 2011; Ying et al., 2003).

**Fig. 4.**
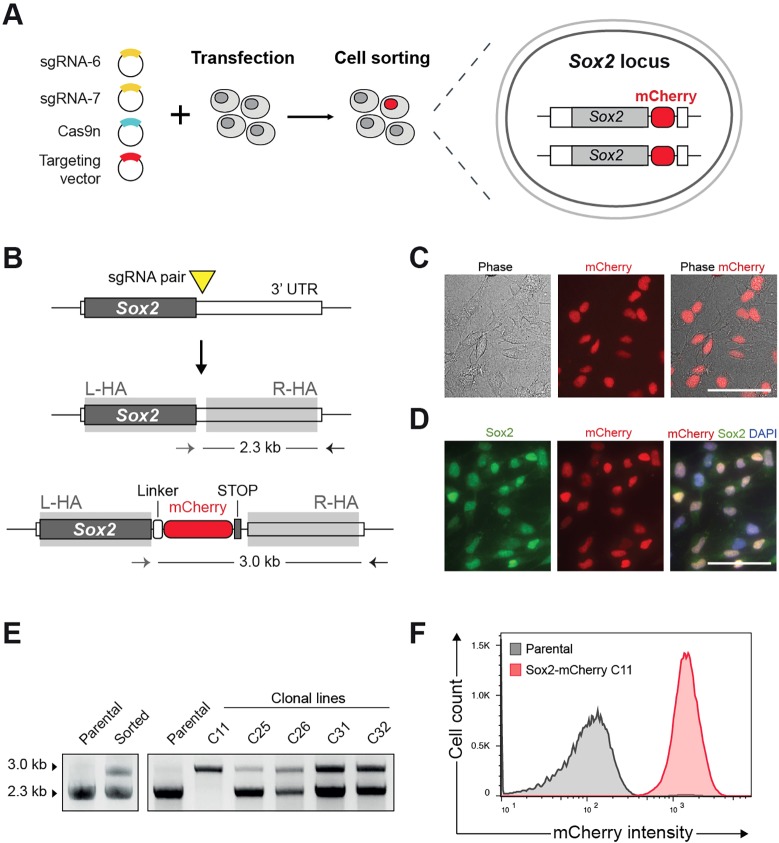
**Generation of homozygous Sox2-mCherry reporter NSC line.** (A) Schematic of the experimental strategy for knock-in of mCherry reporter at the *Sox2* C terminus. Cells were transfected with the targeting vector together with sgRNA pair and Cas9n plasmids. After 10 days, mCherry-positive cells were isolated by cell sorting. (B) Representation of *Sox2* targeted locus and PCR-based genotyping. No promoter sequence is contained within the targeting vector (promoterless construct) and therefore mCherry expression is expected only when the *Sox2* locus is correctly targeted. sgRNA pair was designed to cut in the 3′ UTR close to the stop codon. mCherry was fused to the *Sox2* coding sequence through a flexible peptide linker (white box). (C) Live images of sorted cells showing nuclear localised mCherry signal. Scale bar: 50 µm. (D) ICC reveals overlap of mCherry and Sox2 in the nucleus. Scale bar: 50 µm. (E) PCR genotyping of clonal lines derived after cell sorting. Homozygous clone (C11) shows only an upper band (3.0 kb), whereas heterozygously targeted clones also show lower, wild-type band (2.3 kb). (F) Flow cytometry histogram confirming consistent mCherry expression in homozygous Sox2-mCherry clonal line. Parental NSCs (grey line) were used to set the gates.

To test whether fluorescent reporters could be knocked in using CRISPR/Cas9-assisted gene targeting in mouse NSCs, we focused on *Sox2*. We designed an antibiotic-selection free strategy in which an mCherry fluorescent reporter cassette, plus a small flexible linker, is introduced at the *Sox2* C terminus, creating a new Sox2-mCherry fusion protein product (Fig. 4A). The targeting vector was promoterless, and consequently mCherry signal can only arise from the correct, in-frame insertion at the endogenous *Sox2* locus.

Cells were transfected with the targeting vector together with the Cas9n and a pair of sgRNAs that cut in the 3′UTR sequence adjacent to the stop codon (sgRNAs 5-6). After 10 days, ∼1% of cells were identified as mCherry positive and isolated by FACS (Fig. S10A). Wide-field immunofluorescence microscopy confirmed nuclear localisation of the mCherry in live cells; this colocalised with Sox2 antibody staining by ICC (Fig. 4C,D; Fig. S10B). Clonal lines were subsequently derived from the sorted mCherry-positive subpopulation and PCR-genotyped (Fig. 4E). From 23 clones screened, 20 were correctly targeted at the *Sox2* locus (∼87% targeting efficiency). Notably, biallelic targeting to create homozygous reporters was achieved with high efficiency (∼26%; *n*=6/23). Flow cytometry analysis confirmed uniform mCherry expression in a homozygous targeted *Sox2*-mCherry NSC clonal line (Fig. 4F). This proliferated normally, displayed NSC morphology and nestin expression and had a diploid karyotype (Fig. S10C,D). Together, these results demonstrate the power of Cas9-assisted HR for facile knock-in of fluorescent protein reporters at endogenous genes in NSCs, and highlight the value of promoterless targeting vectors to isolate biallelic targeted cells.

### Efficient knock-in of epitope tags using single-stranded oligonucleotide donor templates

Epitope tagging involves fusion of a small peptide (e.g. V5, FLAG, HA or MYC) to the protein of interest. This simplifies immunoprecipitation, immunoblotting and immunocytochemistry, and is highly desirable when good quality antibodies are unavailable. The approach, however, has been mainly limited to ectopically expressed transgenes, with the attendant limitations of their non-physiological levels of expression. Knock in of epitope tags to endogenous genes therefore represents a more attractive experimental approach and has been widely employed in model organisms, such as *Saccharomyces cerevisiae*, in which HR is highly efficient (Ghaemmaghami et al., 2003). Recently, this has also been reported in mammalian ESC and cancer cell lines through CRISPR/Cas9-assisted gene editing (Savic et al., 2015; Yang et al., 2013). We therefore assessed whether knock-in of epitope tags to endogenous genes could be achieved in mouse NSC lines and developed optimised protocols for this purpose.

Because of the reduced length of coding sequences for epitope tags, we opted to use short oligodeoxynucleotides as a donor template, thus avoiding the need for targeting vector production. Initially, we attempted to insert a V5 tag into *Olig2* and *Sox2* using a strategy previously described in human ESC cultures (Ran et al., 2013). This relies on NHEJ-based insertion of double-stranded oligodeoxynucleotide (dsODN) donors containing overhangs compatible with Cas9n-induced double nicking. Although ICC identified clear V5 nuclear staining in ∼1.5% of cells, unfortunately this strategy was undermined by the persistent emergence of undesirable flanking indels in the resulting clonal lines (data not shown).

**Fig. 5.**
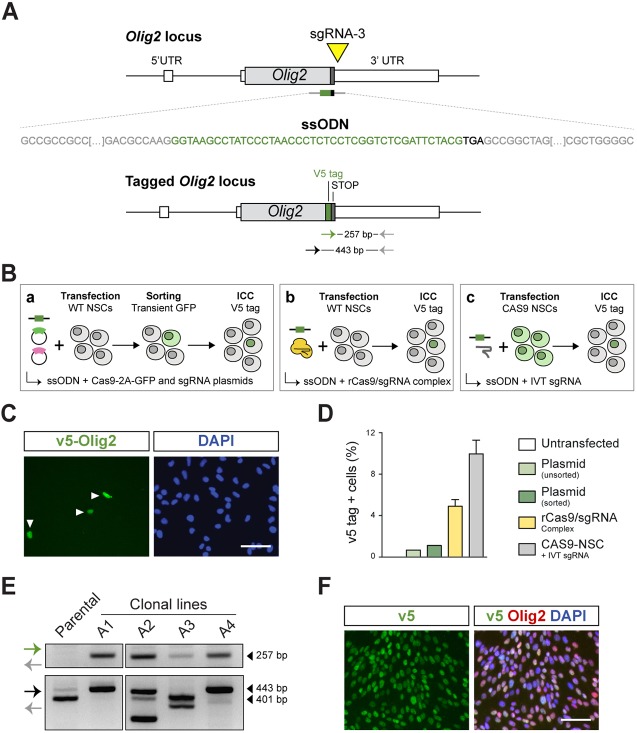
**CRISPR/Cas9-based gene targeting enables epitope tagging of endogenous transcription factor genes.** (A) Representation of strategy to knock in the V5 tag in-frame into the Olig2 C terminus using a single-stranded oligonucleotide DNA (ssODN) as a donor template. V5 tag sequence is shown in green, homology arms in grey and stop codon in black. Yellow triangle represents the sgRNA target site (sgRNA-3) at *Olig2* 3′UTR. (B) Schematic depicting the three strategies employed for the knock-in of the V5 epitope tag: (a) delivery of plasmids encoding sgRNA and Cas9-2A-GFP followed by FACS enrichment; (b) delivery of a ribonucleoprotein complex made by conjugating *in vitro* transcribed (IVT) sgRNA and recombinant Cas9 protein (rCas9); (c) delivery of IVT sgRNA into the CAS9 NSCs (constitute Cas9 expressing from Rosa26). (C) Representative image of V5-tagged Olig2 cells (arrowheads) identified using ICC against the V5 tag. DAPI was used for nuclear staining. Scale bar: 50 µm. (D) Quantification of Olig2-V5-positive cells using the three delivery strategies (shown in B). Values represent the percentage of V5-positive cells relative to total DAPI nuclear counting. (E) PCR-based genotyping of representative v5-Olig2 clones derived from the bulk cells transfected with rCas9+IVT sgRNA complex. PCR1 used primers within the V5 sequence and outside the R-HA. Homozygosity was confirmed using primers flanking the V5 insertion site (PCR2). Homozygous targeted clones (A1 and A4) were identified by a single upper band 42 bp higher than the control, WT band. Clones displaying two bands were considered as heterozygous. (F) ICC for the V5 tag and Olig2 in homozygously tagged clonal lines. As anticipated, V5 staining is nuclear localised and overlaps with native Olig2 staining. Scale bar: 50 µm.

A simpler alternative strategy was therefore pursued, wherein single-stranded ∼185-nt long oligodeoxynucleotides (ssODN) were used as repair templates. These comprised 45 nucleotides of a V5 tag sequence flanked by 70-nt long homology arms. To avoid disruption of endogenous gene-coding sequences, gRNAs were designed to target the 3′UTR region proximal to the stop codons (Fig. 5A). The protospacer adjacent motif (PAM) sequences contained in the ssODN repair templates were altered to prevent re-cutting of the edited alleles. The knock-in efficiencies were quantified by ICC with a V5-tag antibody and three distinct delivery strategies were assessed: (1) transient Cas9- and sgRNA-coding plasmids; (2) *in vitro* transcribed (IVT) sgRNA complexed with recombinant Cas9 protein (rCas9-sgRNA complex); (3) delivery of IVT sgRNA into the Cas9-expressing NSCs (Fig. 5B). For the delivery of recombinant Cas9 and/or IVT sgRNA, we optimised the transfection protocol using the Amaxa 4D system (Fig. S11A,B).

Highest levels of knock-in (∼10%) were achieved using the CAS9-NSCs by transfection of only IVT gRNAs and ssODNs (Fig. 5C,D), whereas plasmid delivery of all components into wild-type cells was much lower. However, ∼5% *Olig2* V5-positive cells were achieved when using the pre-assembled rCas9/sgRNA complex (>5-fold greater than plasmid delivery). This is efficient enough that clonal lines could be established, and offers greater flexibility then using the CAS9-NSCs, as any existing NSC line can be engineered with this approach.

To determine the precise status of each allele, clonal lines were derived from the bulk population and PCR genotyped (Fig. 5E). Two out of five V5-positive clones were found to be tagged at each allele, demonstrating the remarkable power of this approach to quickly isolate clones with biallelicly tagged proteins. Sanger sequencing of the PCR products confirmed correct, in-frame insertion of the V5 tag sequence into Olig2 C-terminus (Fig. S11C), and ICC indicated complete colocalisation of the V5 tag and Olig2 signals in the established clonal lines (Fig. 5F).

Successful knock-in of the V5 tag was also achieved at *Sox2* locus using both the rCas9/sgRNA complex and CAS9-NSCs (∼1.5 and 5.5%, respectively) (Fig. S11D). Similar tagging efficiencies were also observed in the primary mouse foetal FNS2 line and in a glioma-initiating line harbouring Ink4a (Cdkn2a, encoding Ink4a/ARF) and EGFR overexpression (Bruggeman et al., 2007) (Fig. S11E). Altogether, these results demonstrate that epitope tags can be effectively knocked into mouse NSC lines without recourse to any selectable markers, targeting vectors or cell sorting.

### Engineering of glioma driver mutations into human NSCs

**Fig. 6.**
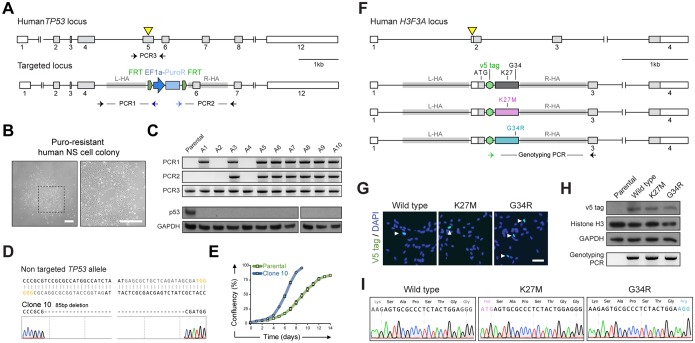
**Delivery of glioma-relevant mutations into genetically normal human NSCs.** (A) Schematic depiction of the targeting strategy to knock out *TP53* via gene targeting. CRISPR sgRNA pair targeting site is indicated with a yellow triangle. Horizontal arrows indicate PCR genotyping primers for assaying *TP53* locus targeting (PCR1 and PCR2) and presence of an indel within the second allele (PCR3). (B) Representative phase contrast images of puromycin-resistant human NSC colony after 10 days of selection. Right-hand image is a magnification of the boxed area on the left. Scale bar: 100 µm. (C) PCR-based genotyping (top) and WB analysis (bottom) of human *TP53* targeted NSC clonal lines. Parental, non-transfected cells were used as a control. (D) Sanger sequencing trace of an exemplar correctly targeted clone harbouring an 85 bp deletion on the second allele. (E) Growth curve analysis of the *TP53* targeted clones harbouring the 85 bp deletion confirming the positive proliferative effect of p53 ablation. (F) Illustration of the strategy for gene targeting the first *H3F3A* coding exon. Yellow triangle indicates sgRNA pair targeting site. Horizontal arrows indicate PCR genotyping primers. (G) ICC using a V5 tag-specific antibody to identify targeted cells (arrowheads). DAPI was used for nuclear staining. Scale bar: 50 µm. (H) Western blotting using V5 tag and histone H3-specific antibodies and PCR genotyping of parental and transfected cells. (I) Sanger sequencing traces of the genotyping PCR products confirm presence of the point mutations in the V5 tag-positive cells.

Glioblastoma stem cells display molecular and phenotypic similarities to NSCs and are thought to arise from endogenous CNS stem or progenitor cells (Stiles and Rowitch, 2008). Thus, engineering glioma mutations in a stepwise manner or in combination into genetically normal human NSCs is an attractive possibility that could open up significant new opportunities for studying mechanisms involved in brain tumour initiation, growth and evolution. Building upon the successful demonstration of gene targeting at the *AAVS1* locus (Fig. 1), we next explored whether disease-relevant knockouts and introduction of point mutations were possible (Fig. 6).

To demonstrate possibilities for loss-of-function mutations, we focused on *TP53*, one of the most frequently mutated tumour suppressors in human cancers, including the majority of GBMs (Brennan et al., 2013). We used the same exon-replacement strategy as described for the mouse NSC lines, in which a vector containing 1 kb long homology arms flanking the Ef1α-PuroR cassette was used to target *TP53* exon 5 (which encodes the DNA-binding domain of p53) (Fig. 6A). Human NSCs (U3 line) were transfected with the targeting vector, a pair of sgRNAs and Cas9n plasmids, and selected 5 days post-transfection. Human NSCs proliferate more slowly than mouse NSCs, which makes genetic manipulations more time-consuming. Nevertheless, puromycin-resistant colonies emerged (Fig. 6B) and clonal lines could be established over a period of 4-6 weeks. PCR genotyping of the clonal lines confirmed successful targeting of one *TP53* allele by the selection cassette in ∼67% of the cases (*n*=8/12; Fig. 6C). Importantly, Sanger sequencing showed the presence of frame-shifting indels in the non-targeted *TP53* allele in the majority of correctly targeted clones (an exemplar clone is shown; Fig. 6D). Similar high-knockout efficiencies were also achieved for two independent human foetal NSC lines (U5 and U3 Hind, not shown). All correctly targeted clones lacked detectable p53 protein levels by WB and ICC analysis (Fig. 6C; Fig. S12). As anticipated, increased cell proliferation was observed following *TP53* knockout (Fig. 6E).

To illustrate engineering of candidate gain-of-function oncogenic mutations, we pursued the delivery of somatic point mutations affecting *H3F3A*, which encodes the histone variant H3.3 and is frequently mutated in paediatric gliomas. Such mutations occur within the N-terminal tail of the histone H3.3 and result in single amino acid substitutions (either K27M or G34R/V) (Schwartzentruber et al., 2012; Wu et al., 2012). From a practical standpoint, the proximity of the mutations to the N terminus proved useful, as we could test if the mutant alleles could be introduced to the endogenous gene using the V5 tag as a reporter of successful knock-in. For this purpose, we used a pair of sgRNAs targeting the 5′UTR region directly upstream of the start codon together with a targeting vector containing 1 kb long homology arms flanking the N-terminal tagged versions of the first coding exon (wild type, K27M or G34R) (Fig. 6F). Targeting efficiencies varied from 0.2 to 0.5% as determined by ICC for the V5 tag (Fig. 6G), with similar efficiencies being achieved for the other two independent human cell lines (U5 and U3 Hind, not shown). WB using antibodies against the V5 tag and histone H3-specific antibodies confirmed the expected size of the tagged protein (around 17 kDa) (Fig. 6H). Correct targeting at *H3F3A* was demonstrated by PCR-based genotyping using primers lying within the V5 tag sequence and downstream of the right homology arm (Fig. 6H). Additionally, Sanger sequencing of the PCR product confirmed the presence of the expected point mutations in the targeted cells (Fig. 6I).

## DISCUSSION

New tools and strategies that enable precise genetic manipulations in NSCs are highly desirable and would open up considerable new opportunities for discovery across stem cell biology, regenerative medicine, neuroscience and related fields. However, targeted genetic manipulations using HR – the mainstay of functional genetic analysis of pluripotent cells – has been technically challenging in most somatic stem cells, largely owing to practical constraints limiting the ability to deliver, isolate and clonally expand cells with the desired genetic change. We reasoned that NSC lines might be particularly well suited to targeted genetic manipulations, as they are readily transfectable and can be clonally expanded in adherent and feeder-free culture conditions. Indeed, we demonstrated here that mouse and human NSC lines are highly amenable to a range of precise genetic manipulations facilitated by the CRISPR/Cas9 technology.

To date, the simplest application of CRISPR/Cas9 in mammalian cells, including mouse neural precursors *in vivo*, has been to disrupt gene function through the generation of random indel mutations (Chen et al., 2015; Kalebic et al., 2016). This is clearly an important application and is particularly well suited to genome-wide screening, as demonstrated recently in human NSC cultures (Toledo et al., 2015). We confirmed that this works well for mouse NSC lines using transient plasmid delivery of CRISPR/Cas9 components followed by FACS-based enrichment of transfected cells. NHEJ-mediated gene disruption was also highly efficient when using the mouse Rosa26-Cas9 NSC line (CAS9-NS), which avoids the need for FACS and has particular value for genetic screenings using pooled libraries of CRISPR sgRNAs, as previously achieved in mouse ESCs (Koike-Yusa et al., 2013).

However, the obvious limitations of gene disruption via indel formation is the lack of control over the types of mutations that emerge and a requirement for screening large numbers of clones to identify the desired loss-of-function mutants. Following the demonstration of transgene insertion via HR at safe harbour loci, we deemed it important to explore whether gene disruption in NSC lines could be achieved via gene targeting, as originally developed in mouse ESCs (Capecchi, 2005). Using an exon-replacement approach recently devised for human iPSCs (W.C.S., unpublished), we demonstrate that mouse NSC lines are highly amenable to gene knockout via HR, with truly remarkable efficiencies of biallelic knockout possible.

Although we did not observe biallelic targeting – i.e. both alleles undergoing successful replacement by HR – we did consistently observe monoallelicly targeted clones harbouring indel mutations on the non-targeted allele. Although deemed disadvantageous under certain circumstances (e.g. knock-in of fluorescent reporters; Merkle et al., 2015), the presence of these indel mutations has considerable practical value as it enables a single round of targeting and selection to virtually guarantee isolation of loss-of-function mutant clones. Moreover, owing to the random nature of the indels, this offers a platform for simple generation of partial loss-of-function (hypomorphic), or, less frequently, gain-of-function (e.g. dominant-negative) alleles in a controllable manner. As only one allele is left after the HR event (i.e. cells are hemizygous), studying the resulting allelic series of distinct clonal lines can offer valuable insights into specific residues or domains of interest. Indeed, sgRNAs might be specifically designed with this purpose in mind, e.g. to probe protein or DNA interaction domains, phosphorylation sites, etc.

To open up possibilities for higher throughput production of mutants, we successfully optimised the transfection protocols, scaling down the amount of cells and DNA used. Remarkably, mutation of 11 transcription factors in NSCs was achieved in only a few weeks (plus ∼2 weeks for targeting vector construction) using a single round of 16 transfections in parallel. From a practical standpoint, this means typically only a handful of NSC colonies need to be picked and screened to isolate biallelic mutants, thereby significantly accelerating precise genetic modification of NSCs. These findings are particularly timely, as the plummeting costs of DNA synthesis now enables rapid production of bespoke and elaborate targeting vectors in weeks rather than months. Furthermore, direct engineering of NSCs is advantageous, as it sidesteps the need for ESC or iPSC modification and subsequent differentiation, which can be laborious and problematic, particularly if the gene of interest is required earlier in development.

Our optimised protocols for efficient gene targeting in mouse NSC lines led us to attempt the more challenging production of knock-in alleles. We focused on generation of fusions of either fluorescent proteins or peptide epitope tags. The knock-in of a fluorescent reporter for *Sox2* – a highly expressed gene in NSCs – was remarkably efficient, highlighting the value of flow cytometry and use of promoterless targeting vectors to enrich biallelic targeted cells. Monitoring protein levels and localisation in live cells using endogenous regulatory elements is therefore now possible and will greatly facilitate functional studies. Complementary epitope tagging of endogenous genes can now simplify protein interaction studies and mapping of genome-bound sites using immunoprecipitation in combination with mass spectrometry (IP-MS) or chromatin immunoprecipitation combined with sequencing (ChIP-Seq), respectively. This will be a massive boost for investigators who are frequently hampered by the limitations of poor quality, non-specific or unavailable antibodies. We found that such knock-in of epitope tags can be rapidly implemented in NSCs, and our data suggest that delivery is most efficient using recombinant Cas9 protein/gRNA complex into NSC cultures. Notably, efficiencies are high enough that cell sorting or drug selection strategies are not required.

The ability to manipulate the NSC genome precisely also has great potential in neuro-oncology. There is now a clear opportunity to engineer candidate driver mutations or epimutations uncovered in genome sequencing projects. We illustrated this by successful mutation of the endogenous *TP53* and *H3F3A* loci in genetically normal human NSC lines. It should now be possible to test the effects of glioma mutations, step-wise and in combination, across a range of spatially and temporally diverse NSCs cultures; and vice versa, efficient gene targeting could now enable gene correction of putative driver mutations in patient-derived GBM cell lines. Together, both approaches create useful isogenic panels of human cellular models for brain tumour research – an important requirement for effective cell-based phenotypic screening.

Risks of off-target mutations with CRISPR are often discussed (Tsai and Joung, 2016). However, for *in vitro* cell lines – as opposed to genome editing in embryos – potential off-target mutations are less of a concern, as one can generate multiple clones using different sgRNAs and also readily perform genetic rescue to provide confidence in any newly identified cellular phenotype. When unique sgRNAs are unavailable, then use of Cas9 nickase reduces the risk of off-target mutations (Cho et al., 2014). Nonetheless, the most recent studies exploring CRISPR-Cas9 off-target effects have not identified significant issues when sgRNAs with unique targets are used (Kim et al., 2016) and therefore initial fears of widespread off-target damage have now waned.

The CRISPR/Cas9 technology has rapidly transformed possibilities for mammalian functional genetics. We can finally move beyond transformed cell lines such as HeLa (Hyman and Simons, 2011) into a new era of systematic functional gene annotation, with multiple genes or pathways being explored in parallel in relevant primary cells. In summary, our findings indicate that targeted, precise and complex genetic manipulations can be readily performed in NSC lines. Genetic control of NSC self-renewal can now investigated using elegant genetic strategies that have been so successful in understanding ESCs (Martello and Smith, 2014). This opens up a wealth of opportunities to explore gene function in CNS development, adult homeostasis and pathological processes.

## MATERIALS AND METHODS

### Design and construction of CRISPR sgRNAs and targeting vectors

CRISPR sgRNAs were designed using the Optimized CRISPR Design tool (http://crispr.mit.edu). Predicted sgRNA off-target sites were retrospectively analysed using the WTSI Genome Editing tool (http://www.sanger.ac.uk/htgt/wge). All sgRNAs were predicted to target unique genomic sites. For the majority of those, similar sequences contained mismatches of three or more nucleotides (with at least one occurring in the PAM proximal region), and therefore off-target cleavage is unlikely (Cho et al., 2014). Sequences are provided in Table S1.

For sgRNA-encoding plasmids, single-stranded oligonucleotides (Integrated DNA Technologies) containing the guide sequence of the sgRNAs were annealed, phosphorylated and ligated into *Bsa*I site of U6-*Bsa*I-sgRNA backbone (kindly provided by S. Gerety, Sanger Institute, Cambridge, UK). For *in vitro* transcription (IVT), dsDNA templates for T7-driven transcription were generated by annealing two oligonucleotides – one containing the T7 promoter and guide RNA target sequences, and the other containing the Cas9-binding tracrRNA sequence. The annealed oligo pair was gap-filled using T4 DNA polymerase, column-purified, and then used as a template for IVT (MEGAscript T7 Transcription Kit). The RNA was purified using MEGAclear Transcription Clean-Up Kit.

Targeting vectors were constructed via Gibson assembly and Gateway cloning (Fig. S3). Briefly, linearised backbone and a Zeo/PheS bacterial selection cassette were obtained through *Eco*RV digestion of existing plasmids. Homology arms of ∼1 kb were amplified from genomic DNA using PCR primers with 22 bp overhangs compatible with both backbone and the Zeo/PheS double-selection cassette. Gibson reactions were performed using a standard protocol with home-made enzyme mix (Gibson et al., 2009) to create the intermediate Gateway cloning compatible intermediate vector. The Zeo/PheS cassette was replaced via LR Gateway cloning using a FRT-Ef1a-PuroR-FRT mammalian selection cassette. For *AAVS1* and *Rosa26* targeting vectors, the Luc-2A-GFP, Cas9-2A-GFP or rtTA expression cassettes were PCR amplified from existing plasmids (gift from M. Pule, University College London, UK) and cloned into Gateway pDONR221 using BP cloning. The cassettes were then delivery into the intermediate targeting vectors via Gateway LR cloning. Construction of the *AAVS1*-TRE-GFP targeting vector involved restriction digestion followed by ligation of a custom gene vector (Life Technologies) containing the TRE-GFP-2A-PuroR cassette into the digested *AAVS1* intermediate targeting vector (Fig. S3). For mouse *Sox2*-mCherry targeting vector, 1 kb long arms were PCR amplified and tethered to mCherry sequence using Gibson reaction. The sgRNA targeting region was removed from the R-HA in targeting vector to avoid re-cutting of residual Cas9 after the homologous recombination event. For *H3F3A* targeting vectors, homology arms were amplified from genomic DNA and Gibson-assembled with synthetic DNA fragments (Life Technologies) containing the V5-tagged, mutant sequences of the first coding exon.

### Plasmid encoded- and recombinant Cas9

Human codon-optimised *Streptococcus pyogenes* wild-type Cas9 (Cas9-2A-GFP) and Cas9 nickase (Cas9n-2A-GFP) plasmids were obtained from Addgene (#44719 and #44720). Recombinant wild-type Cas9 was purchased from PNA Bio.

### Cell culture

Mouse adult NS line (ANS4) was derived from the subventricular zone as previously described (Pollard et al., 2006). Mouse foetal NSC lines FNS2 and PG1-1 were derived from the telencephalon region of embryonic day 11.5 and 17.5 embryos, respectively. Human lines were derived from the telencephalon of ∼8-week-old human fetal material. The human fetal material used to derive U3 and U5 cell lines was provided by the Joint MRC/Wellcome Trust (grant number 099175/Z/12/Z) Human Developmental Biology Resource (www.hdbr.org). Tissue was donated with informed consent after elective termination of pregnancy. Detailed protocols for derivation of mouse and human foetal cell lines are described elsewhere (Conti et al., 2005; Sun et al., 2008). RNA-seq profiling of human NSCs confirmed expression of NS affiliated lineage marker. Genomic profiling of these lines using GeneChip SNP 6.0 arrays (Affymetrix) did not identify any structural genetic alterations (not shown).

Mouse and human lines were expanded for more than 15 passages before use in gene-targeting experiments. Established lines were propagated in serum-free basal medium supplemented with N2 and B27 (Life Technologies), laminin (Sigma, 1 µg/ml) and growth factors EGF and FGF2 (Peprotech, 10 ng/ml). Medium was changed every 2-3 days and cells split 1:3 or 1:5 once per week after dissociation with Accutase solution (Sigma). Laminin was added directly to the culture media, with no need for pre-coating of flasks. This improved colony formation and simplified screening. Colonies were picked by manual aspiration using a 20 µl pipette. Confluence analysis and growth curves were determined using Incucyte (Essen Bioscience) live cell imaging system.

### Cell transfection

Cells were transfected using either Amaxa 2B or 4D nucleofection systems (Lonza) according to the manufacturer's instructions. For the 2B system, 2×10^6^ cells were pre-mixed with plasmid DNA, 2 µg of the indicated Cas9 vector, 1 µg of each sgRNA and 1 µg of the targeting vector or 2 µg of ssODN in 100 µl of Neural Stem Cell Amaxa nucleofection buffer. Nucleofection program T-030 and X-005 was used for mouse and human NSCs, respectively. For the 4D system, 16-strip cuvettes were loaded with, unless otherwise stated, 4×10^5^ cells and 0.8 µg plasmid DNA (0.4 µg Cas9, 0.1 µg each sgRNA and 0.2 µg targeting vector) in SG transfection solution (Lonza). Program DN100 gave best survival and transfection efficiency for plasmid DNA delivery. For delivery of the Cas9/sgRNA complex, 5 µg (unless otherwise stated) of recombinant Cas9 were mixed with 3 µg of *in vitro*-transcribed sgRNA and allowed to form ribonucleoprotein complex at room temperature for 10 min. The Cas9/sgRNA complex together with 1.5 µg of ssODN was transfected into 2×10^5^ cells using the Amaxa 4D 16-strip cuvettes in SG transfection buffer. Program EN138 gave the best results for rCas9/IVT sgRNA delivery. After transfection, cells were recovered in pre-warmed culture media and plated onto 10 cm culture dishes for 5 days prior to drug selection or downstream assays.

### T7 endonuclease I assay

Genomic regions flanking the CRISPR sgRNA target sites were PCR amplified using gene-specific primers (Table S2). PCR products were purified with MinElute PCR Purification Kit (Qiagen) and hybridised in NEB buffer 2 (95°C, 5 min; 95-85°C at −2°C/s; 85-25°C at −0.1°C/s; hold at 4°C). After treatment with T7 endonuclease I (5 U, NEB) at 37°C for 1 h, the resulting fragments were subjected to electrophoresis in a 2.5% agarose gel and visualised by staining with ethidium bromide. Cleavage quantification was based on relative band intensities using ImageJ.

### Immunocytochemistry

Cells were fixed in 4% paraformaldehyde, permeabilised and blocked in 0.1% bovine serum albumin plus 3% goat serum solution. Samples were incubated overnight with primary antibodies followed by incubation with appropriate secondary antibodies (1:1000; Invitrogen) and 4′,6-diamidino-2-phenylindole (DAPI). Immunopositive cells were quantified using ∼5000 cells (minimum of ten random fields). Total cell number was determined by DAPI nuclear staining. The following primary antibodies were used: mouse nestin (1:10; Developmental Studies Hybridoma Bank, Rat-401), human nestin (1:500; R&D Systems, MAB1259), mouse Sox2 (1:100; Abcam, 92494), human Sox2 (1:50; R&D Systems, MAB2018), BLBP (also known as Fabp7) (1:200; Santa Cruz, sc-30088), GFAP (1:1000; Sigma, G3893), Tuj1 (also known as Tubb3) (1:1000; Biolegend, 801202), GFP (1:1000; Abcam, AB13970), Olig2 (1:400; Millipore, AB9610), mCherry (1:500; Abcam, AB167453), V5 tag (1:1000; eBioscience, 14679682), p53 (1:400; Cell Signaling, 2524).

### Western immunoblotting

Immunoblotting was performed using standard protocols. Antibodies were diluted in 5% milk powder in TBS-T, and protein detection was carried out with horseradish peroxidase-coupled secondary antibodies (ThermoFisher Scientific, A16110 and A16072) and exposed to X-ray film. The following primary antibodies were used: Olig2 (1:3000; Millipore, AB9610), GAPDH (1:1000; GenTex, GTX627408), p53 (1:500; Cell Signaling, 2524), V5 tag (1:1000; eBioscience, 14679682); histone H3 (1:2000; Abcam, AB24834).

### Drug selection and clonal expansion

Cells with stable targeting vector integration were selected using 5 µg/ml blastidicin or 0.1 µg/ml puromycin. Selection commenced 5 days post-nucleofection to enable time for expression of Cas9, HR and expression of selection cassettes. After 7-10 days of blasticidin selection, *Rosa26* and *AAVS1* targeted cells were enriched by FACS (GFP expression) and plated at <5000 cells/10 cm dish for recovery of colonies. For the knockout experiments, cells were kept in puromycin for 7 days. Resistant colonies were individually picked into 2×24 or 96-well replica plates and expanded for cell banking and DNA extraction. Cells for genomic DNA extraction were cultured until >90% confluent.

### PCR-based genotyping of targeted clones

Genomic DNA was isolated from each well of a confluent 24- or 96-well plate as follows: cells were incubated for 2 h at 55°C in 20 or 40 μl of lysis buffer (0.45% NP40, 0.45% Tween20, 1× NEB LongAmp PCR buffer) and subsequently heated to 95°C (10 min). One to two microlitres of this lysate was used in a 10 μl PCR reaction. PCR reactions comprised 0.2 μl DMSO (100% v/v, Sigma), 0.3 μl dNTPs (10 mM, Thermo Fisher Scientific), 2.0 μl LongAMP buffer (5× NEB), 0.4 μl LongAMP Taq (NEB) and 12 pmol of each primer. Thermal cycling was performed using the following conditions: 1 cycle 94°C for 3 min; 40 cycles 94°C for 15 s, 60°C for 30 s, 65°C for 2 min; followed by final extension at 65°C for 10 min.

For each targeted locus, two sets of genotyping primers spanning the junction of genomic sequences and targeting vector were used (left and right arms). Gene-specific primers were designed outside the 5′ and 3′ homology arms and were used in combination with primers in the knock-in cassette (either CAG-LUC-2A-GFP-IRES-BSD for targeting *Rosa26* and *AAVS1* loci, or Ef1α-Puromycin for the knockout experiments). To identify NHEJ-based indel formation on the second, non-targeted alleles, the region flanking the sgRNA target sites (500-600 bp) was amplified using PCR with gene-specific primers and directly assessed by Sanger sequencing. Sequences of all primers are provided (Table S2).

### qPCR copy number analysis

Quantitative PCR with the TaqMan Copy Number Assay (Applied Biosystems) was performed as previously described (Schick et al., 2016). A custom Puromycin-resistance gene (PuroR) probe was used with reference *Tfrc* (4458367). Genomic DNA from a mouse ESC line harbouring a single genomic copy of GFP-IRES-PuroR (TNG; gift from Ian Chambers, University of Edinburgh, UK) was used as calibrator sample.

### qRT-PCR

RNA was extracted using the RNeasy spin column kit (Qiagen), plus DNase treatment to eliminate gDNA. cDNA was generated with SuperScript III (Invitrogen), and quantitative RT-PCR was performed using Taqman Universal PCR Master Mix (Applied Biosystems). The following Taqman assays (Life Technologies) were used: mOlig2 (Mm01210556_m1), mGAPDH (Mm99999915_g1), hTP53 (Hs01034249_m1) and hGAPDH (Hs02758991_g1).

### Metaphase spread

For karyotyping, cells were treated with nocodazole (5 h) and metaphase spreads prepared as previously described (Campos et al., 2009). Modal chromosomal number of the parental and clonal lines was determined by counting chromosomes of at least 20 mitotic cells.

### Differentiation assay

Glial and neuronal differentiation was initiated by removing EGF from the culture media for 3 or 7 days (mouse and human, respectively). Cells were then allowed to differentiate in the presence of 1% foetal calf serum for an additional 3 or 14 days. For differentiation of mouse PG1-1 cells into oligodendrocyte precursors, EGF was removed from the media for 4 days.
